# Natriuretic Peptides for the Detection of Paroxysmal Atrial Fibrillation in Patients with Cerebral Ischemia – the Find-AF Study

**DOI:** 10.1371/journal.pone.0034351

**Published:** 2012-04-11

**Authors:** Rolf Wachter, Rosine Lahno, Beatrice Haase, Mark Weber-Krüger, Joachim Seegers, Frank Edelmann, Janin Wohlfahrt, Götz Gelbrich, Anke Görlitz, Pawel Kermer, Dirk Vollmann, Gerd Hasenfuß, Klaus Gröschel, Raoul Stahrenberg

**Affiliations:** 1 Department of Cardiology and Pneumology, University of Göttingen, Göttingen, Germany; 2 Department of Neurology, University of Göttingen, Göttingen, Germany; 3 Center for Clinical Trials, University of Leipzig, Leipzig, Germany; 4 Institute for Clinical Trials, University of Göttingen, Göttingen, Germany; 5 Department of Neurology, University of Mainz, Mainz, Germany; Inserm, France

## Abstract

**Background and Purpose:**

Diagnosis of paroxysmal atrial fibrillation (AF) can be challenging, but it is highly relevant in patients presenting with sinus rhythm and acute cerebral ischemia. We aimed to evaluate prospectively whether natriuretic peptide levels and kinetics identify patients with paroxysmal AF.

**Methods:**

Patients with acute cerebral ischemia were included into the prospective observational Find-AF study. N-terminal pro brain-type natriuretic peptide (NT-proBNP), brain-type natriuretic peptide (BNP) and N-terminal pro atrial-type natriuretic peptide (NT-proANP) plasma levels were measured on admission, after 6 and 24 hours. Patients free from AF at presentation received 7 day Holter monitoring. We prospectively hypothesized that patients presenting in sinus rhythm with NT-proBNP>median were more likely to have paroxysmal AF than patients with NT-proBNP<median.

**Results:**

281 patients were included, of whom 237 (84.3%) presented in sinus rhythm. 220 patients naïve to AF with an evaluable prolonged Holter ECG were analysed. In patients with NT-proBNP>median (239 pg/ml), 17.9% had paroxysmal AF in contrast to 7.4% with NT-proBNP<239 pg/ml (p = 0.025). The ratio of early (0 h) to late (24 h) plasma levels of NT-proBNP showed no difference between both groups. For the detection of paroxysmal atrial fibrillation, BNP, NT-proBNP and NT-proANP at admission had an area under the curve in ROC analysis of 0.747 (0.663–0.831), 0.638 (0.531–0.744) and 0.663 (0.566–0.761), respectively. In multivariate analysis, BNP was the only biomarker to be independently predictive for paroxysmal atrial fibrillation.

**Conclusions:**

BNP is independently predictive of paroxysmal AF detected by prolonged ECG monitoring in patients with cerebral ischemia and may be used to effectively select patients for prolonged Holter monitoring.

## Introduction

Atrial fibrillation (AF) is a frequent cause of ischemic stroke and patients with atrial fibrillation bear an increased risk for suffering a recurrent stroke [1], [2]. Diagnosis of AF in patients with ischemic stroke usually results in a change of therapy with oral anticoagulation being the most effective strategy for secondary prevention of ischemic events [3], [4]. Identification of paroxysmal AF after cerebral ischemia and initiation of appropriate therapy can therefore be expected to lower morbidity from recurrent stroke. Unfortunately, patients with paroxysmal AF may be asymptomatic until the occurrence of a cerebral embolic event. Identifying paroxysmal AF may be challenging in these subjects if they are in sinus rhythm at the time of hospitalisation. Prolonged [5] or continued [6] rhythm monitoring may enhance the detection of clinically ‘silent’ paroxysmal AF, but being expensive, time-consuming in terms of evaluation as well as cumbersome for the patient, broad application of such enhanced diagnostics may not be readily available for all unselected patients presenting with cerebral ischemia of unknown cause and sinus rhythm on admission.

Natriuretic peptides have been widely used in different clinical settings and have been shown to be predictive of AF within the general population [7] as well as in patients undergoing surgical procedures [8]. Moreover, numerous studies have shown an elevation of natriuretic peptide levels in AF [9], [10] which decrease after conversion [11], [12] to sinus rhythm. Thus, we hypothesized that natriuretic peptide levels as well as the ratio of early (0 h) to late (24 h) plasma level of a natriuretic peptide may identify acute stroke patients with paroxysmal AF.

## Materials and Methods

### Patients

Find-AF is a single center prospective observational trial (ISRCTN 46104198). Details on patient recruitment and detection rate of Holter monitoring have recently been published [5]. The present analysis aimed to determine whether a blood test (plasma levels of natriuretic peptides) can help risk stratification in patients with cerebral ischemia to further more intensive diagnostics (7 d-Holter ECG). We included consecutive patients presenting to the emergency department of the University of Göttingen between March 2009 and February 2010 with symptoms of stroke or transient ischemic attack (TIA). Patients who were found to have other definitive diagnoses (e.g. intracranial bleeding) causative for their symptoms were excluded, all others were asked to confirm preliminary consent by signature. Exclusion criteria were age <18 years or inability or unwillingness to consent. The study complies with the Declaration of Helsinki, the protocol was approved by the responsible ethics committee of the University of Göttingen and all patients gave written informed consent.

### Data collection and clinical evaluation

Baseline characteristics were recorded by a standardised questionnaire, including detailed medical history and baseline medication. Patients underwent serial biomarker sampling at 0 h, 6 h and 24 h after presentation to the emergency department and received carotid ultrasound as well as cerebral imaging (computed tomography or magnetic resonance imaging). After written informed consent was obtained, patients without AF at baseline received a Holter monitor (CardioMem® CM 3000, getemed Medizin- und Informationstechnik, Teltow, Germany) which was applied by specifically trained study personnel (R.C.L. and B.H.). These devices are capable of recording a period of up to 10 days with a single charge of batteries on a secure digital storage card. Patients and relatives were instructed in the correct handling of the monitors. The Holter monitors were collected after 7 days. Patients discharged earlier were instructed to send back the devices after the monitoring period had elapsed.

Plasma levels of NT-proBNP were measured using a sandwich enzyme immunoassay (Roche Diagnostics, Mannheim, Germany). NT-proANP was analysed with an ELISA (Biomedica, Wien, Austria). BNP was measured by means of a sandwich chemiluminescence immunoassay on a Centaur (Bayer Vital, Leverkusen, Germany). Personnel responsible for the determination of natriuretic peptide levels were blinded to clinical patient data.

### Definition of the primary endpoint

The primary hypothesis of Find-AF was that higher NT-proBNP levels on admission and thus can discriminate patients with paroxysmal AF from those without AF. As natriuretic peptides have been shown to fall rapidly after conversion of AF to sinus rhythm, we also investigated the ratio of early (0 h) to late (24 h) plasma levels of NT-proBNP. Furthermore, we measured both BNP (which is measured in many institutions instead of NT-proBNP), and NT-proANP, which is a more stable precursor of atrial natriuretic peptide and may be more specific for atrial hemodynamic stress than the BNPs.

We assumed that 20% of the patients with NT-proBNP above median and 5% of those with NT-proBNP below median had paroxysmal AF [13]. To detect a difference with a two-sided α = 0.05 and power of 90%, 101 study participants per group (202 overall) would be necessary. Considering a rate of 10% of ECGs not evaluable and drop-outs, we planned to include 225 patients.

### Definitions and Statistical Analyses

Continuous data are given as mean ± standard deviation, unless otherwise stated. Categorical variables are given as absolute numbers (percent) and were compared by Chi-square or Fisher's exact test. Normally distributed data were compared by student's t test, not normally distributed data by Mann-Whitney-U-Test. To investigate relations between variables, bivariate Pearson's correlations were calculated. To elucidate the independency of association with paroxysmal AF, we performed multivariate analyses with age, sex, body mass index, systolic blood pressure, estimated glomerular filtration rate (eGFR), coronary artery disease, presence of heart failure, left atrial diameter, left ventricular ejection fraction, left ventricular mass index and left ventricular enddiastolic diameter as covariables and added natriuretic peptide plasma levels on admission or 0/24 h ratios individually to the base model. Receiver operating characteristics (ROC) curves were used to describe test characteristics. Etiology of stroke was classified according to the widely used TOAST classification scheme and was performed by one of the neurologists (J.W. or K.G.). Stroke severity was approximated by video trained physicians applying the NIH stroke scale (NIH-SS) and the modified Rankin Scale (mRS). Presence of paroxysmal AF was defined as at least one period of more than 30 seconds duration of an absolute arrhythmia without detectable P waves and without a pattern more consistent with an alternative diagnosis, as recommended and described previously [5]. Detected episodes were verified by a specialist in electrophysiology (J.S.) blinded to clinical data and natriuretic peptide levels. The analysis included only those 220 patients who were naive to AF and had an evaluable Holter ECG. Statistical tests were performed with SPSS Statistics 19.0.0 (IBM, Chicago, Illinois, USA). Values of natriuretic peptides were not normally distributed and were thus logarithmised for correlation and logistic regression analysis.

## Results

### Study population

**Figure 1:**
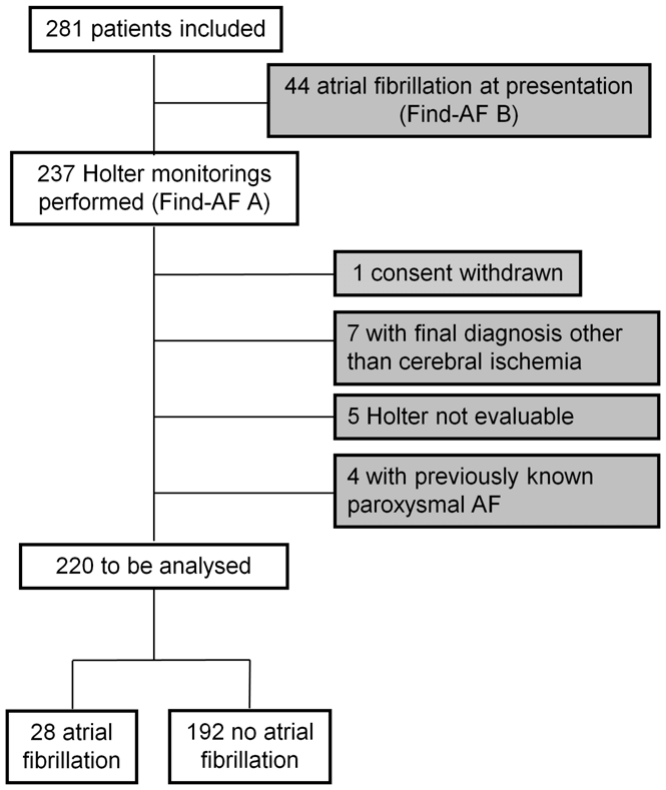
Study flow of the Find-AF trial. 281 patients were included, 237 had sinus rhythm on the admission ECG. 1 patient withdrew consent, 16 patients were excluded from the analysis for different reasons, given a total of 220 study participants analysed.

281 consecutive patients were included, of whom 44 (15.7%) had AF at presentation. All remaining patients (n = 237) underwent Holter monitoring. Clinical characteristics of the complete cohort have previously been reported [5]. One patient withdrew consent. For the present analysis, we excluded patients with a final diagnosis other than cerebral ischemia (n = 7), those with Holter ECGs that could not be analysed (n = 5) and those with previously diagnosed paroxysmal AF (n = 4), see also figure 1. Of the remaining 220 patients, 28 (12.7%) had paroxysmal AF (n = 26) or atrial flutter (n = 2) on Holter monitoring. Table 1 shows clinical characteristics of the 28 patients with paroxysmal AF and the 192 patients without paroxysmal AF. The median CHADS_2_ score in patients with paroxysmal AF was 3.8.

**Table 1:** Patient characteristics.

		No paroxysmal atrial fibrillation(n = 192)	Paroxysmal atrial fibrillation(n = 28)	p
Age		67±13	76±12	0.001
Female gender		80 (41.7%)	12 (42.9%)	0.905
BMI		27.5±5.7	26.8±4.7	0.484
NIH-SS		3±4	7±4	<0.001
Modified Rankin Scale		2±1	3±1	<0.001
Stroke severity†	TIA	68 (35.4%)	2 (7.1%)	0.001
	Minor stroke	52 (27.1%)	6 (21.4%)	
	Major stroke	72 (37.5%)	20 (71.4%)	
TOAST classification	Large artery atherosclerosis	39 (20.3%)	0 (0.0%)	0.008
	Cardioembolic	28 (14.6%)	11 (39.3%)	
	Lacunar/small vessels	27 (14.1%)	0 (0.0%)	
	Rare/other causes	5 (2.6%)	0 (0.0%)	
	Undetermined cause	93 (48.4%)	17 (60.7%)	
Heart rate		72±13	71±16	0.656
Systolic blood pressure		143±22	154±26	0.010
Diastolic blood pressure		79±13	80±14	0.489
Creatinine [mg/dl]		1.0±0.5	1.0±0.5	0.616
Estimated glomerular filtration rate (ml/min/1,73 m^2^)		84±26	76±26	0.133
Hemoglobin [mg/dl]		13.9±1.7	13.2±1.8	0.033
TSH [iU/ml]		2.6±11.3	2.2±3.2	0.864
Left atrial diameter [mm]		41±7	44±6	0.050
Left ventricular ejection fraction [%]		62±9	59±10	0.309
Left ventricular mass index [g/m^2^]		219±79	222±59	0.857
History of stroke		29 (15.1%)	5 (17.9%)	0.707
History of TIA		18 (9.4%)	2 (7.1%)	0.701
Heart failure		10 (5.2%)	2 (7.1%)	0.674
Hypertension		136 (70.8%)	23 (82.1%)	0.212
Diabetes		43 (22.4%)	7 (25.0%)	0.759
Smoker		51 (26.6%)	4 (14.3%)	0.062
Hyperlipidemia		60 (31.3%)	14 (50.0%)	0.056
Coronary artery disease		22 (11.5%)	11 (39.3%)	0.001
Peripheral artery disease		5 (2.6%)	2 (7.1%)	0.201
Medication				
ACE inhibitors/ARBs		106 (55.2%)	15 (53.6%)	0.968
Beta-blockers		71 (37.0%)	16 (57.1%)	0.067
Calcium antagonists		34 (17.7%)	2 (7.1%)	0.255
Statins		46 (24.0%)	12 (42.9%)	0.059
Antiarrhythmics		2 (1.0%)	0 (0.0%)	0.601

† Minor stroke resolved completely within 30 days or change in NIH stroke scale by 3 points; major stroke neurologic deficit persisted after 30 days and increased NIH stroke scale by 3 points.

ARB: Angiotensin receptor blocker.

### Natriuretic peptides and paroxysmal atrial fibrillation

**Figure 2:**
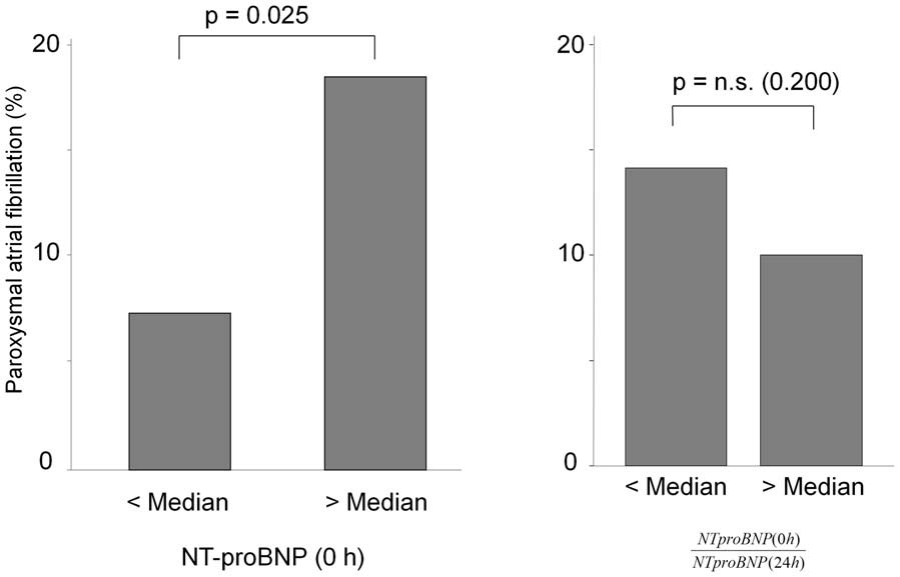
NT-proBNP and NT-proBNP ratio and paroxysmal atrial fibrillation. Left panel: Percentage of study participants with paroxysmal atrial fibrillation on Holter monitoring in the two sub-groups of patients with NT-proBNP plasma levels below and above the median NT-proBNP plasma level (239 pg/ml). Right panel: Percentage of study participants with paroxysmal atrial fibrillation below and above the median ratio of early (0 h) to late (24 h) NT-proBNP plasma level (0.78).

Table 2 shows levels of NT-proBNP, BNP and NT-proANP at three different time-points (0 h, 6 h, 24 h) as well as the ratio of early (0 h) to late (24 h) plasma levels. All natriuretic peptides were significantly higher in patients with paroxysmal AF at each time point, but the ratio of early to late plasma levels were not different between those with and without paroxysmal atrial fibrillation. The median NT-proBNP level at 0 h was 239 pg/ml; the median ratio of early to late plasma NT-proBNP-level was 0.78. Figure 2 shows the primary parameters of the study: The prevalence of paroxysmal AF in study participants with a NT-proBNP above the median was 17.9% compared to 7.4% in patients below the median NT-proBNP (p = 0.025). The prevalence of paroxysmal AF between study participants with a ratio of early/late NT-proBNP above median was not different to those with a ratio below median (p = 0.200). Of note, BNP and NT-proBNP both correlated with NIH-SS (r = 0.196, p = 0.010 and r = 0.176, p = 0.014, respectively) and mRS (r = 0.248, p = 0.001 and r = 0.257, p<0.001, respectively), whereas log NT-proANP did neither correlate with NIH-SS (r = 0.103, p = 0.153) nor with mRS (r = 0.139, p = 0.054). All natriuretic peptides correlated with age (r = 0.417, r = 0.231 and r = 0.373 for BNP, NT-proANP and NT-proBNP, respectively, all p≤0.001). Similar correlations were found for BNP and NT-proANP.

**Table 2:** Natriuretic peptides plasma levels at three different timepoints and the ratio of early (0 h) to late (24 h) natriuretic peptide level.

	n =	No paroxysmal atrial fibrillation(n = 192)	Paroxysmal atrial fibrillation(n = 28)	P value
NT-proBNP 0 h (pg/ml)	201	195 (74–564)	467 (212–872)	0.023
NT-proBNP 6 h (pg/ml)	192	260 (90–883)	635 (256–1334)	0.029
NT-proBNP 24 h (pg/ml)	207	276 (94–902)	983 (297–1785)	0.004
Ratio (NT-proBNP 0 h/NT-proBNP 24 h)	188	0.79 (0.48–1.09)	0.68 (0.38–0.88)	0.078
BNP 0 h (pg/ml)	178	104 (50–189)	181 (133–384)	<0.001
BNP 6 h (pg/ml)	181	108 (53–251)	321 (170–564)	<0.001
BNP 24 h (pg/ml)	194	116 (46–253)	336 (188–838)	<0.001
Ratio (BNP 0 h/BNP 24 h)	156	0.93 (0.63–1.26)	0.64 (0.43–0.96)	0.027
NT-proANP 0 h (pg/ml)	197	84000 (46291–128778)	114206 (95142–163170)	0.007
NT-proANP 6 h (pg/ml)	190	79774 (40607–116863)	102715 (82224–132688)	0.029
NT-proANP 24 h (pg/ml)	202	68199 (43400–118676)	118379 (77422–140828)	0.007
Ratio (NT-proANP 0 h/NT-proANP 24 h)	180	1.20 (0.90–1.50)	1.24 (0.90–1.62)	0.708

All data are displayed as median (25%–75% percentile). Data are compared by Mann-Whitney-U-Test.

None of the natriuretic peptides showed a significant correlation with the cumulative time in AF during the 7-day Holter ECG. Of note, natriuretic peptides between patients with paroxysmal atrial fibrillation and the 44 patients with AF on admission were not significantly different.

### Diagnostic utility of natriuretic peptides

**Figure 3:**
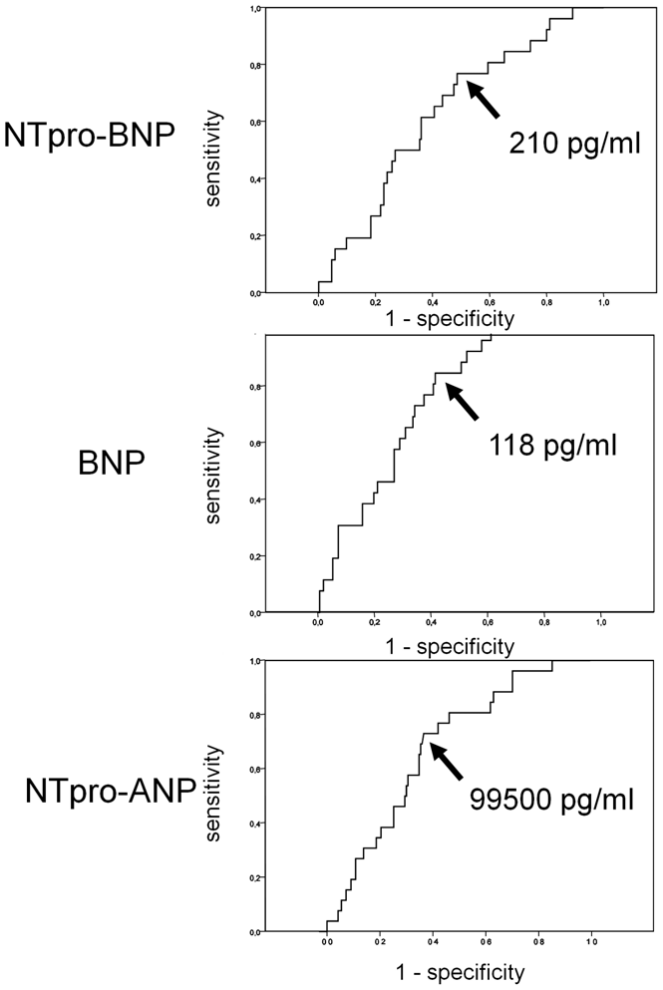
Diagnostic properties of natriuretic peptide levels for detecting paroxysmal atrial fibrillation. Upper panel: Receiver operating characteristic curve for NT-proBNP plasma levels in the detection of paroxysmal atrial fibrillation. The arrow indicates the Youden-point, which was a NT-proBNP plasma level of 210 pg/ml. Middle panel: Receiver operating characteristic curve for BNP plasma levels in the detection of paroxysmal atrial fibrillation. The arrow indicates the Youden-point, which was a BNP plasma level of 118 pg/ml. Lower panel: Receiver operating characteristic curve for NT-proANP plasma levels in the detection of paroxysmal atrial fibrillation. The arrow indicates the Youden-point, which was a NT-proANP plasma level of 99,500 pg/ml.

We performed receiver-operating characteristic analysis to determine the diagnostic utility of natriuretic peptides for the detection of paroxysmal AF. Figure 3 shows the ROC curves for NT-proBNP, BNP and NT-proANP plasma levels on admission. The area under the curve was 0.638 (95% CI: 0.531–0.744) for NT-proBNP, 0.747 (95% CI: 0.663–0.831) for BNP and 0.663 (95% CI: 0.566–0.761) for NT-proANP. The combination of two different natriuretic peptides did not show incremental value. Table 3 shows test characteristics for different BNP cut-offs.

**Table 3:** Test characteristics for different BNP cut-offs and corresponding sensitivity, specificity, positive and negative predictive values and the number of patients needing Holter.

BNP (pg/ml)Cut-off	Sensitivity	Specificity	PPV	NPV	Needing Holter
86	96%	42%	22%	98%	63%
99	92%	47%	23%	97%	58%
118*	85%	59%	26%	96%	48%
134	73%	63%	25%	93%	43%

PPV: Positive predictive value. NPV: Negative predictive value.

* denotes the Youden point.

As patients with systolic heart failure have elevated NT-proBNP plasma levels, we performed a subgroup analysis and excluded all patients with an ejection fraction <50% (n = 15). This did not substantially alter the results. In multivariate analyses with age, sex, body mass index, systolic blood pressure, estimated glomerular filtration rate, coronary artery disease and presence of heart failure as covariables, baseline BNP was the only natriuretic peptide to be independently predictive for the presence of paroxysmal AF on 7 d Holter monitoring (corrected odd's ratio 6.1 per ten-fold BNP, 95% CI 1.7 to 22.4, p = 0.006).

## Discussion

To the best of our knowledge this study tested for the first time prospectively a biomarker-guided approach to predict paroxysmal AF in patients with acute cerebral ischemia and sinus rhythm at the time of initial presentation.

Our study has three major findings:

1. Natriuretic peptide plasma levels are significantly higher in patients with paroxysmal AF compared to those without.
2. The kinetics of natriuretic plasma levels within the first 24 h after admission do not differ between patients with paroxysmal AF and those without.
3. BNP was independently predictive for paroxysmal AF as detected by highly sensitive 7 d Holter monitoring while NTproBNP and NTproANP were not.

Natriuretic peptides have been shown to be predictive for future AF in a number of different clinical settings [8], [14], including the general population [7], [15]. However, no previous study has evaluated its usefulness for the detection of paroxysmal AF. Our results are to some extent in line with previous data presented in a recent work of Okada et al. [16]. These authors found elevated BNP in stroke patients who had sinus rhythm on admission, but developed AF during follow-up. Within our cohort, the prevalence of paroxysmal AF is nearly three times higher in patients with NT-proBNP above the median of 239 pg/ml compared to those with NT-proBNP below median. However, the discriminatory value of all natriuretic peptides for the detection of patients with paroxysmal AF was only moderate (best area under the curve 0.747 for BNP) which limits the value of these biomarkers in patients with cerebral ischemia. We found similar results for biomarkers in the detection of paroxysmal AF in patients with hypertension, but without prior stroke (unpublished data). BNP was the only natriuretic peptide to be predictive for paroxysmal AF in multivariate analyses. This discrepancy between previous reports of natriuretic peptides as predictors of future atrial fibrillation and elevated levels in persistent atrial fibrillation may well be explained by the population studied and our study design: To reflect a real-life stroke scenario, we included an unselected cohort of patients with cerebral ischemia, rather heterogeneous and with many comorbidities. This may increase variability in biomarker plasma levels due to cofactors. A second factor that may have increased variability in natriuretic peptide levels not due to AF may have been its very early measurement in the emergency department. As a stroke may be associated with acute hemodynamic perturbations, natriuretic peptide levels might be altered by the index event itself. However, only an unselected cohort like the one included in Find-AF can be truly informative about the value of a diagnostic method in clinical practice. Another important factor may be the use of 7 d Holter monitoring for the diagnosis of paroxysmal AF. Because this method is more sensitive to detect paroxysmal AF in these patients, we probably identified patients with a lower burden of AF than in most other studies. Especially asymptomatic patients and those with only short episodes of AF may experience less hemodynamic compromise and therefore have lower natriuretic peptide levels than those with longer episodes or severe symptoms that allow a clinical diagnosis in studies with less sensitive diagnostic methods.

Also of theoretic interest is the fact that the ratio of early to late natriuretic peptide levels was not different between patients with and without AF, although it has convincingly been demonstrated that the main cardiac source of natriuretic peptide release in AF are the atria [17] and that the plasma levels fall after conversion from AF to sinus rhythm [12]. Two mechanisms may explain this finding:

1. NT-proBNP increased from baseline to 6 and 24 hours after the index event in all patients irrespective of paroxysmal AF. This may be explained by a release of NT-proBNP reflecting a vasodilating response to the cerebral ischemic event or a direct myocardial dysfunction [18]. Patients with paroxysmal AF had larger strokes (median NIH stroke scale 7 vs. 3) and had a higher morbidity (mRS 3 vs. 2). Thus, a decrease in NT-proBNP released from the heart may have been counterbalanced by an increase in NT-proBNP released from brain tissue resulting in a lack of difference in NT-proBNP between the two groups. However, the absence of a difference in NT-proANP levels argues against this hypothesis.
2. The time difference between conversion to sinus rhythm and the cerebral ischemic events was longer than assumed. Earlier studies have shown that it takes up to 4 weeks after electric cardioversion for a restoration of atrial contraction [19] and that the risk for thromboembolism is increased in patients without anticoagulation especially within the first days after conversion from AF to sinus rhythm. If the conversion took place several days before the index event (cerebral ischemia), there might be no relevant decrease in NT-proBNP plasma levels in the first 24 hours after the index event.

### Strengths and limitations

Our study is strengthened by its size and the prospective evaluation of natriuretic peptide levels as a primary objective. Moreover, the use and detailed analysis of 7 day-Holter ECG for the evaluation of paroxysmal AF allowed detecting a high number of affected patients, superior to periodic ECGs and cardiac event loop recorders [20]. However, we can not exclude that a larger number of patients with paroxysmal AF may have been detected (e. g. longer duration of Holter ECG monitoring or implantable loop recorders).

### Clinical implications

The most relevant complication of untreated AF is cerebral ischemia and systemic embolism and it has convincingly been shown that this risk is similar in paroxysmal and sustained (persistent or permanent) AF [21]. Patients with cerebral ischemia and AF have an indication for oral anticoagulation, independent of AF type, and oral anticoagulation is capable to cut down the risk for recurrent stroke by nearly 2/3 [22]. Thus, the identification of an increased risk for paroxysmal AF by a natriuretic peptide is highly attractive and clinically relevant in patients presenting with acute cerebral ischemia and sinus rhythm. We have previously shown that 7 d Holter monitoring increases the diagnostic yield for paroxysmal AF in these patients. Our data show that BNP is the only independent predictor of paroxysmal AF. If only patients with a BNP>99 pg/ml would have been screened by 7 day Holter ECG, this would have reduced the number of patients needing 7 day Holter ECG by 42% with only 8% of patients with paroxysmal AF being missed.

### Summary

Although natriuretic peptide levels are higher in stroke patients with paroxysmal AF compared to patients with permanent sinus rhythm, our prospective investigation shows that BNP is the only natriuretic peptide independently predictive of paroxysmal atrial fibrillation. BNP may therefore be used as a rapidly available tool for early risk stratification in patients with cerebral ischemia.
